# Liquorice Intoxication Can Lead to Cardiac Arrest!

**DOI:** 10.1155/2020/3727682

**Published:** 2020-09-21

**Authors:** Rachid Attou, Sébastien Redant, Patrick M. Honore, Thierry Preseau, Philippe Hantson, David De Bels

**Affiliations:** ^1^Intensive Care Department, Brugmann University Hospital, Brussels, Belgium; ^2^Emergency Department, Brugmann University Hospital, Brussels, Belgium; ^3^Intensive Care Department, Cliniques Universitaires St-Luc, Brussels, Belgium; ^4^Louvain Centre for Toxicology and Applied Pharmacology, Université catholique de Louvain, Brussels, Belgium

## Abstract

A 45-year-old man was admitted to the Emergency Department with fatigue and muscular weakness. Soon after hospital admission, he developed “torsades de pointe” and was successfully resuscitated. The admission laboratory investigations had revealed a profound hypokalemia (1.65 mmol/L). The patient had a long-term use of alcohol-free “pastis” in an attempt to reduce his chronic ethanol consumption. As the beverage likely contained a significant amount of liquorice, the diagnosis of glycyrrhizin chronic intoxication was suspected. The diagnosis of liquorice-related pseudohyperaldosteronism was assessed by normal plasma aldosterone levels and low plasma renin activity. Intravenous and oral supplementation of potassium was required for 5 days, and the patient had an uneventful follow-up.

## 1. Introduction

Liquorice root (*Glycyrrhiza gabra*) is one of the oldest plants used in some traditional medicines. The major bioactive principle is glycyrrhizic acid which has been shown to inhibit (after intestinal hydrolysis to glycyrrhetic acid) the enzyme 11-*β*-hydroxysteroid dehydrogenase. This could result to pseudohyperaldosteronism with hypokalemia, hypertension, and metabolic alkalosis. Liquorice is widely present in food and additives leading to a possible chronic intoxication. We describe an unusual case complicated by “torsades de pointe” and cardiac arrest following the chronic ingestion of an alcohol-free “pastis” beverage.

## 2. Case Report

A 45-year-old Caucasian man presented to our Emergency Department for asthenia, muscular weakness, polydipsia, and severe hypertension. His medical history revealed arterial hypertension and chronic alcohol consumption. He was currently treated with amlodipine, perindopril, and vitamin B supplementation. According to the relatives, the patient ingested daily a large amount of an alcohol-free “pastis” beverage in an attempt to reduce ethanol consumption. At physical examination, arterial blood pressure was 240/120 mmHg, heart rate 75/min cardiac and pulmonary auscultation was normal, and a mild lower limb edema was noted. Admission laboratory investigations are summarized in Table 1 and were remarkable for hypokalemia (1.65 mmol/L, Nl 3.5-5). Liver function tests were nearly normal with AST 42 UI/L (nl < 40), ALT 38 UI/L (nl < 41), alacaline phosphate 115 UI/L (nl 40-129), and gamma GT 102 UI/L (nl 10-71). Total bilirubin was 0.7 mg/dL (nl < 1.1). Fifteen minutes after hospital admissions, the patient experienced an episode of “torsades de pointe” rapidly evolving to cardiac arrest. Short (2 min) cardiopulmonary resuscitation included a 200 J asynchrone defibrillation and the administration of 1 mg of intravenous epinephrine. Postcardiac arrest arterial blood gas did not show any severe metabolic acidosis: pH 7.49, pCO_2_ 48 mmhg (nl 35-45), PO_2_ 75 mmHg (nl 75-104), and bicarbonate 32 mmol/L (nl 28-32).

Postcardiac arrest electrocardiogram (ECG) revealed an irregular sinus rhythm associated with diffuse repolarization abnormalities; the QTc interval was 410 msec. The patient was transferred to the ICU for further monitoring and continuous intravenous potassium administration through a central venous line for 2 days. Oral supplementation was started on day 3, and the patient was discharged to the general ward. Hormonal workup revealed normal supine plasma aldosterone levels accompanied by very low plasma renin activity. The patient was discharged four days later. At 6-month follow-up, the patient had a normal blood pressure and kalaemia after having stopped liquorice-based beverages.

## 3. Discussion

Differential diagnosis of severe hypokalemia is very important as it mainly conditions the best therapeutics in ED. It includes decrease intake (starvation, clay ingestion), cell redistribution (metabolic alkalosis; treatments such as insulin, *β*-2-agonists, or *α*-2-antagonists; B_12_ vitamin or folic acid; parenteral nutrition…), and increased loss (mineralocorticoid excess including liquorice or Tobacco chewing, vomiting, renal tubular acidosis, and diabetic keto-acidosis). The patients' vitamin B was without B_12_. High levels of intravenous KCl were administered to him during in ED and ICU stay.

Hypokalemia, of chronic installation, can be very severe and associated with cramps, paresthesia, and heart rate disturbances such as ventricular fibrillation or “torsades de pointe.” Severe hypokalemia can also induce rhabdomyolysis as illustrated in our patient [1].

There was no liver failure as total bilirubin was only very lightly elevated and under the toxic range (0.7 mg/dL, nl < 1.1), but gamma-glutamyltransferase was elevated probably mainly due to chronic alcohol abuse.

Diagnosis can be made by low plasma aldosterone concentration, very low plasma renin levels, and disturbed urinary (Na/K) electrolytes [2]. Hypernatremia and metabolic alkalosis are inconstant [3].

Glycyrrhizin is a terpene extracted from the liquorice root. Natural liquorice juice contains 5 to 20% of glycyrrhizin. It is mainly used as a sweetener, its sweet power being 30 to 50 times greater than saccharose which explains its use in confectionery and beverages such as “pastis” as in the present observation.

Glycyrrhizin intoxication is mainly due to excessive and prolonged consumption of liquorice based pastry or cocktails. Chronic ingestion of 65-165 g liquorice confectionary can still cause symptoms, even if there is probably some interindividual susceptibility to glycyrrhetic acid. In 2005, the Food and Drug Administration (FDA) has implemented the World Health Organization recommendations of maximum glycyrrhizin concentrations in food and beverages [4]. They are illustrated in Table 2.

**Figure 1:**
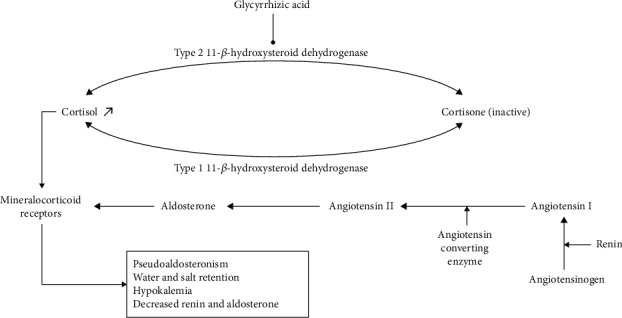
Both cortisol and aldosterone bind with equal affinity to mineralocorticoid receptors that are activated predominantly with aldosterone. Type 2 11-*β*-hydroxysteroid dehydrogenase (11-*β*HSD) converts cortisol to its inactive form, cortisone, and evokes corticosteroid specificity. By inhibitory effects of *Glycyrrhiza glabra* and glycyrrhizin on type 2 11-*β*HSD, the mineralocorticoid receptors are activated by cortisol, leading to accumulation of glucocorticoids with anti-inflammatory and mineralocorticoid properties, hypokalemia, and mineralocorticoid-related hypertension.

Liquorice has a mineralocorticoid action through its glycyrrhetic derivative which inhibits type 2 11-*β*-hydroxysteroid dehydrogenase (Figure 1) and furthers renal transformation of cortisol to cortisone. Cortisol is free to bind to mineralocorticoid receptors mimicking hyperaldosteronism with severe hypokalemia, water, and salt retention [5].

Intoxication is often suspected when arterial hypertension appears to be refractory to medical treatment in patients who are acknowledging liquorice consumption. Efficacy of angiotensin converting enzyme inhibitors is often limited in this setting by the too low plasma renin levels [5, 6].

Glycyrrhizin intoxication may lead in some patients to severe cardiac arrhythmias. In a review on nine patients with severe liquorice intoxication, six developed cardiac arrest [7]. The patients survived after successful resuscitation. Acute heart failure with pulmonary edema has also been described [8].

Evolution is usually favourable within 30 days of liquorice weaning. Mineralocorticosteroid effect can last 6 months because of long glycyrrhizin half-life and the time needed for normalization of renin-angiotensin-aldosterone pathway [4].

## 4. Conclusion

The association of severe hypokalemia and hypertension should draw the attention to a possible liquorice intoxication, in the absence of other evident causes of drug-induced hypokalemia. Thorough patient's history and the determination of renin and aldosterone plasma levels are the cornerstone of the diagnosis. Treatment includes potassium supplementation and spironolactone as well as stopping glycyrrhizin uptake [9, 10].

## Figures and Tables

**Table 1:** Pertinent laboratory findings.

Parameter	Value	Normal value
Plasma		
Plasma sodium	144	135-145 mmol/L
Plasma potassium	1.65	3.5-5 mmol/L
Plasma chloride	101	95-105 mmol/L
Plasma bicarbonate	43	22-29 mmol/L
Plasma creatine kinase	2270	<210 UI/L
Plasma creatinine	0.80	0.70-1.20 mg/dL
Plasma glucose	12	3.3-5.5 mmol/L
Urine		
Urinary sodium	156	mmol/24 h
Urinary potassium	80	mmol/24 h
Plasma renin activity		
Supine	<0.04	0.04-0.52 pmol/L/s
Upright	0.43	0.28-1.06 pmol/L/s
Supine plasma aldosterone	106	22-477 pmol/L

**Table 2:** Limitations for the use of liquorice and its derivatives in foods.

Food category	Maximum allowable levels of glycyrrhizin content in food (%)
Hard candy	16
Soft candy	3.1
Chewing gum	1.1
Vitamins or mineral dietary supplements	0.5
Nonalcoholic beverages	0.15
Herbs and seasonings	0.15
Plant protein products	0.15
Alcoholic beverages	0.10
All other foods except sugar substitutes	0.10
Baked goods	0.05
